# Supplementary material for: Performance and Biomass Characteristics of SBRs Treating High-Salinity Wastewater at Presence of Anionic Surfactants
Source: Int J Environ Res Public Health. 2020 Apr 14;17(8):2689. doi: 10.3390/ijerph17082689 (PMC7216276; doi:10.3390/ijerph17082689)
Supplement: Supplementary file 1 [file ijerph-17-02689-s001.pdf]

Supplementary Material

Performance and Biomass Characteristics of SBRs Treating High-Salinity Wastewater at Presence of Anionic Surfactants

Huiru Li [1], Shaohua Wu [1,2] and Chunping Yang [1,2,3,*]

[1] College of Environmental Science and Engineering, Hunan University and Key Laboratory of Environmental Biology and Pollution Control (Hunan University), Ministry of Education, Changsha, Hunan 410082, China; lihuiru@hnu.edu.cn (H.L.); wushaohua@hnu.edu.cn (S.W.)
[2] Guangdong Provincial Key Laboratory of Petrochemical Pollution Processes and Control, School of Environmental Science and Engineering, Guangdong University of Petrochemical Technology, Maoming, Guangdong 525000, China
[3] Hunan Provincial Environmental Protection Engineering Center for Organic Pollution Control of Urban Water and Wastewater, Changsha, Hunan 410001, China
* Correspondence: yangc@hnu.edu.cn (C.Y.)

Analytical methods

The effluent samples were collected at the end of the second cycle everyday, and then the concentrations of ammonia nitrogen and total phosphorus of samples were measured. The measurement continued until the removal rates of ammonia nitrogen and total phosphorus stabilized. When the removal of nitrogen and phosphorus is stable, samples were taken every half an hour to determine the changes of various indicators during the operation cycle. During the second operation cycle for one day, collect 1 ml sludge mixed liquor sample every half hour for dehydrogenase activity (DHA) detection and collect 10 ml sludge mixed liquor sample for the detection of other indicators simultaneously. These indicators included ammonia nitrogen, total phosphorus, nitrite nitrogen, nitrate nitrogen, protein and polysaccharide. Among them, the sludge mixed liquor samples of 1 mL each time which used for the determination of DHA were detection by TTC staining in accordance with Xia et al. [1]. Another 10 mL sludge mixed liquor samples need to be centrifuged at 8000 r/min for 10 min immediately after collected. The supernatant was collected and used to detect changes in the concentration of ammonia nitrogen, total phosphorus, nitrite nitrogen and nitrate nitrogen. The measuring method of ammonia nitrogen concentration is Nessler reagent colorimetry, the measuring method of total phosphorus concentration is ammonium molybdate spectrophotometry, and Chemical Oxygen Demand (COD) was measured by sealed digestion dichromate method [2-4]. N-(1-nyl)-ethylenediamine spectrophotometry [5] and ultraviolet spectrophotometry [6] were used to determine the concentration of nitrite nitrogen and nitrate nitrogen at different times during the cycle operation. After further processing, the centrifugal sediment was first obtained loosely bound extracellular polymer (LB-EPS) [7], and then tightly bound extracellular polymer (TB-EPS). Using bovine serum albumin as

the standard solution, the content of protein (PN) in the sample was determined by the Coomassie brilliant blue method [8]. The polysaccharide (PS) content is determined by the anthracenone sulfate method with glucose as the standard solution [9]. In addition, when the removal performance of SBR dosed with 0.08g/L SDS and SDBS was stable, sludge samples from three SBRs were taken for 16S rRNA sequencing analysis [2].

16S rRNA sequencing analysis

The Illumina high-throughput sequencing included DNA extraction, followed by PCR amplification using primer pairs 515F and 806R, targeting the V4 region of 16S rRNA gene [10,11]. The 16S rRNA gene amplicon products were sequenced using the HiSeq2500 platform at Illumina. The detailed protocols of these two processes were applied according to Chen et al. [2]. All valid sequences of the three samples were clustered by Uparse software (V7.0.1001), and then the required operational taxa were screened by RDP classifier (V2.2) and against the GreenGene database with a confidence threshold of 80%. Operational taxonomic units (OTUs) clustered at 97% similarity and some other indexes indicating the microbial diversity of samples were presented. Alpha diversity and beta diversity of microbial community were analyzed using QIIME (V1.7.0) and R software (V2.15.3) [12].

1. Xia, X.Q., Lin, S.Y., Zhao, J., Zhang, W., Lin, K.F., Lu, Q., Zhou, B.S., 2018. Toxic responses of microorganisms to nickel exposure in farmland soil in the presence of earthworm (Eisenia fetida). Chemosphere. 192, 43-50. 43.

2. Chen, Y.J., He, H.J., Liu, H.Y., Li, H.R., Zeng, G.M, Xia, X., Yang, C.P., 2018. Effect of salinity on removal performance and activated sludge characteristics in sequencing batch reactors. Bioresource Technol. 249, 890-899.

3. Zhou, Q., Lin, Y., Li, X., Yang, C.P., Han, Z.F., Zeng, G.M., Lu, L., He S.Y., 2018. Effect of zinc ions on nutrient removal and growth of Lemna aequinoctialis from anaerobically digested swine wastewater. Bioresource Technol, 249, 457-463.

4. Zhou, Q., Li, X., Lin, Y., Yang, C.P., Tang, W.C., Wu, S.H., Li, D.H., Lou, W., Effects of copper ions on removal of nutrients from swine wastewater and release of dissolved organic matters in duckweed systems. Water Res, 2019, 158: 171-181.

5. Zhang, Q., Liu, S.J., Yang, C.P., Chen, F.M., Lu, S.L., 2014. Bioreactor consisting of pressurized aeration and dissolved air flotation for domestic wastewater treatment. Sep Purif Technol. 138, 186-190.

6. Zhang, Y., Cheng, Y., Yang, C.P., Luo, W., Zeng, G.M., Lu, L., 2015. Performance of system consisting of vertical flow trickling filter and horizontal flow multi-soil-layering reactor for treatment of rural wastewater. Bioresource Technol. 193, 424-432.

7. Li, X.Y., Yang, S.F., 2007. Influence of loosely bound extracellular polymeric substances (EPS) on the flocculation, sedimentation and dewaterability of activated sludge. Water Res. 41(5), 1022-1030.

8. Hou, W.H., Chen, X., Song, G.L., Wang, Q.H., Chi Chang, C., 2007. Effects of copper and cadmium on heavy metal polluted waterbody restoration by duckweed (Lemna minor). Plant Physiol Bioch. 45(1), 62-69.

9. Cheng, Y., He, H.J., Yang, C.P., Yan, Z., Zeng, G.M., Qian, H., 2016. Effects of anionic surfactant on n-hexane removal in biofilters. Chemosphere. 150, 248-253.

10. Sheel, A., Pant, D., 2018. Recovery of gold from electronic waste using chemical assisted microbial biosorption (hybrid) technique. Bioresource Technol. 247, 1189-1192.

11. Yang, G., Wang, J., 2019. Changes in microbial community structure during

dark fermentative hydrogen production. Int J Hydrogen Energ. 200, 217-222.

12. Haas, B.J., Gevers, D., Earl, A.M., Feldgarden, M., Ward, D.V., Giannoukos, G., Ciulla, D., Tabbaa, D., Highlander, S.K., Sodergren, E., Methe, B., DeSantis, T.Z., Human Microbiome, C., Petrosino, J.F., Knight, R., Birren, B.W. 2011. Chimeric 16S rRNA sequence formation and detection in Sanger and 454-pyrosequenced PCR amplicons. Genome Res. 21(3), 494-504.

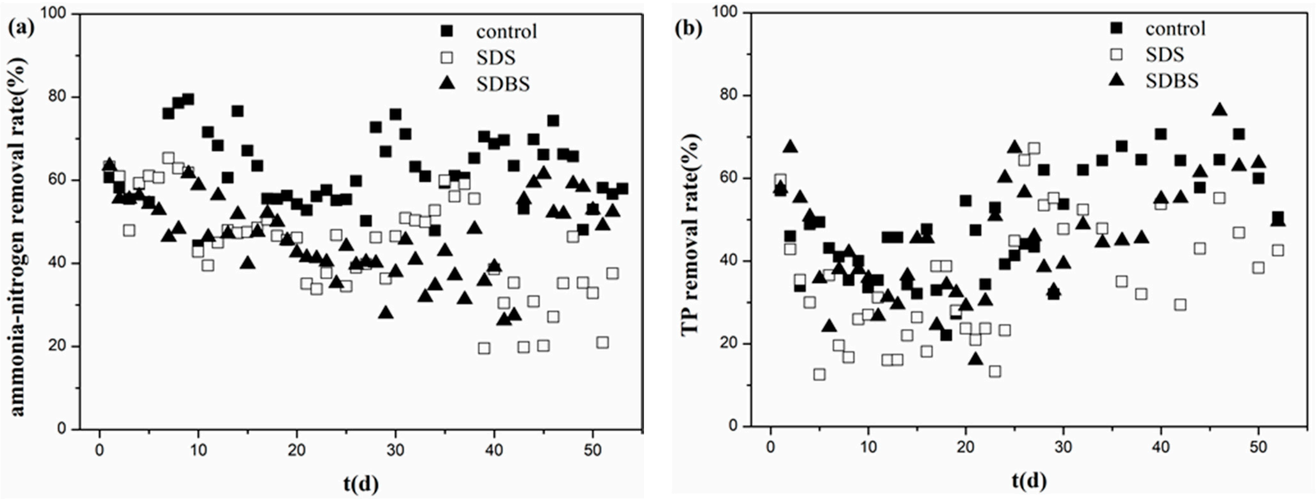

Fig. S.1. Removal rate of pollutants in SBRs by adding surfactant: (a) NH^{4+}-N; (b) TP

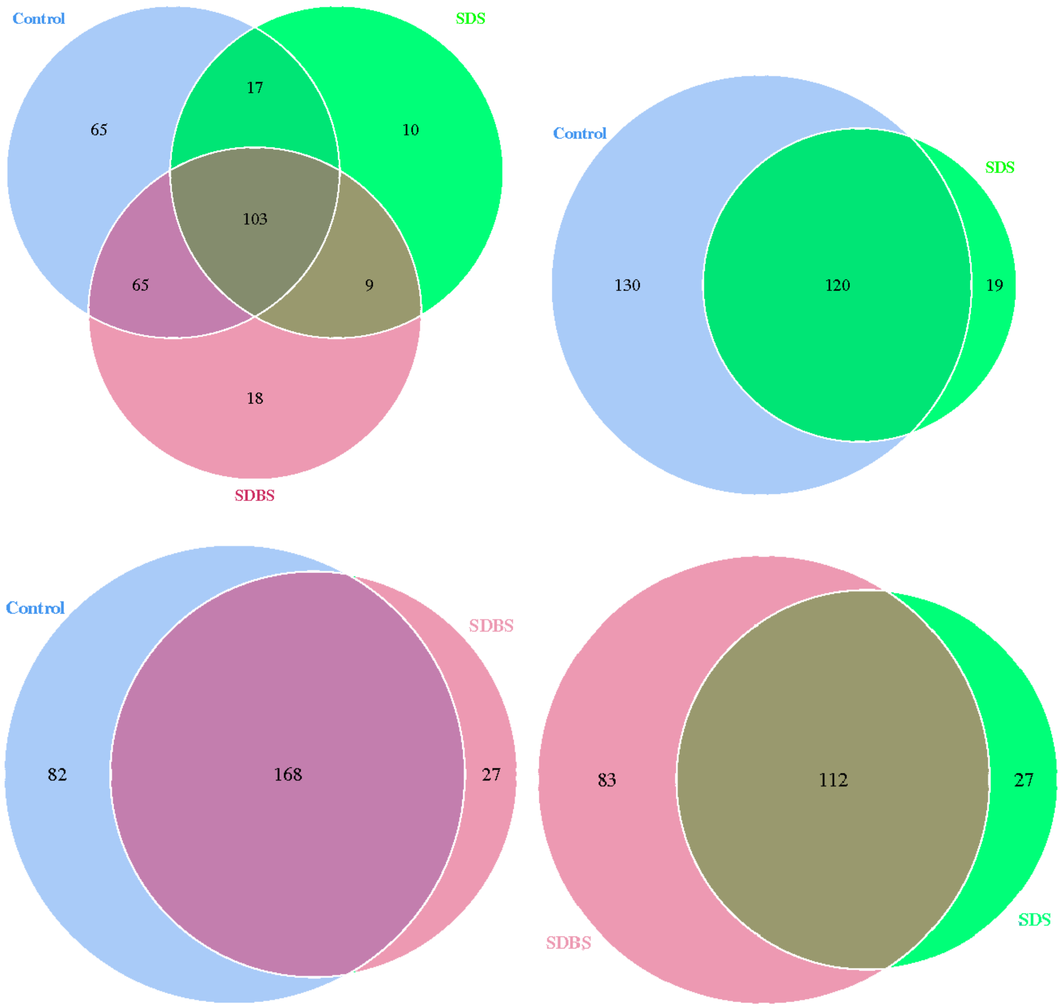

Fig. S.2. The Wayne diagram of the microbial community
